# Integrating single-cell RNA-sequencing and functional assays to decipher mammary cell states and lineage hierarchies

**DOI:** 10.1038/s41523-020-00175-8

**Published:** 2020-07-29

**Authors:** Joseph L. Regan, Matthew J. Smalley

**Affiliations:** 1 Charité Comprehensive Cancer Centre, Charité – Universitätsmedizin Berlin, Charitéplatz 1, 10117 Berlin, Germany; 2 European Cancer Stem Cell Research Institute, School of Biosciences, Cardiff University, Hadyn Ellis Building, Wales, CF24 4HQ UK

**Keywords:** Differentiation, Mammary stem cells, Gene expression, Sequencing, Breast cancer

## Abstract

The identification and molecular characterization of cellular hierarchies in complex tissues is key to understanding both normal cellular homeostasis and tumorigenesis. The mammary epithelium is a heterogeneous tissue consisting of two main cellular compartments, an outer basal layer containing myoepithelial cells and an inner luminal layer consisting of estrogen receptor-negative (ER^−^) ductal cells and secretory alveolar cells (in the fully functional differentiated tissue) and hormone-responsive estrogen receptor-positive (ER^+^) cells. Recent publications have used single-cell RNA-sequencing (scRNA-seq) analysis to decipher epithelial cell differentiation hierarchies in human and murine mammary glands, and reported the identification of new cell types and states based on the expression of the luminal progenitor cell marker KIT (c-Kit). These studies allow for comprehensive and unbiased analysis of the different cell types that constitute a heterogeneous tissue. Here we discuss scRNA-seq studies in the context of previous research in which mammary epithelial cell populations were molecularly and functionally characterized, and identified c-Kit^+^ progenitors and cell states analogous to those reported in the recent scRNA-seq studies.

Previous studies to elucidate the cellular identities of mammary epithelial subpopulations have involved functional and molecular characterization by flow cytometric and functional (down to single cell) transplantation assays^1–14^, as well as, more recently, lineage-tracing studies^15–26^. Transplantation experiments have generally supported a model in which facultative MaSCs, cells capable of regenerating the epithelium when injected into a cleared mammary fat pad (one free of endogenous epithelium)^1,27^, are localized to the basal cell layer^2,5,9,28,29,30^. Progenitor cells, which are functionally defined by high colony-forming and proliferative potential in vitro and limited repopulating ability when transplanted into cleared fat pads, are localized to the luminal layer^6,10,28,29^. Differentiated cells do not transplant or generate colonies in vitro. The molecular profiling of mammary epithelial subpopulations functionally defined by their transplantation potential has been extensive^9,17,31–40^.

Supporting this model, in situ evidence, including lineage-tracing studies from early mammary development, puberty, and alveolargenesis during pregnancy, has shown that basal cells can contribute to the luminal layer^19,41–43^. We previously proposed, based on in situ analysis, that basal MaSCs located in the cap cell layer of terminal end buds (TEBS), the outermost cell layer of the specialized growth structure that drives ductal growth during puberty, are bipotent and produce daughter cells that contribute to both the basal and luminal cell lineages^43^. Lineage-tracing experiments from Rios et al.^16^ and Wang et al.^15^ were in agreement with transplantation data and our in situ analysis, suggesting that MaSCs in the developing postnatal gland are bipotent^15,16,43^. However, more recently, it has been shown that, rather than a transcriptionally defined bipotent TEB MaSC, a group of transcriptionally heterogeneous lineage-committed MaSCs mediate development of the pubertal mammary gland and contribute transiently to ductal expansion^23^, mirroring the organization and neutral drift of adult stem cells observed in the intestine^44,45^. This model of postnatal mammary gland development is in agreement with saturation, single-cell genetic, and neutral lineage-tracing studies demonstrating that bipotent fetal MaSCs (fMaSCs), first functionally and molecularly characterized (including single-cell gene expression analysis demonstrating molecular heterogeneity) by Spike et al.^37^, exist in the embryo, but that in the postnatal gland, basal and luminal lineages are maintained by separate lineage-committed stem/progenitor populations^18–24,42,46–48^. During oncogenic transformation, basal and luminal cell populations may lose this restricted lineage potential and acquire multipotency^20,24,49,50^.

Recent studies have used scRNA-seq, which unlike functional and population-based sequencing studies, allows for unbiased analysis of individual cells in a heterogeneous tissue, to decipher lineage hierarchies and cell states in the mammary epithelium^51–54^. To investigate cellular heterogeneity and lineage relationships in the human breast, Nguyen et al.^51^ performed scRNA-seq analysis on fluorescence-activated cell-sorted (FACS) breast epithelial cells and reported the identification of additional cell types within the three main mammary epithelial cell populations, previously identified as basal (B: CD49f^High^ EPCAM^+^, K14^+^), luminal progenitors (L1: CD49f^+^ EPCAM^+^, ER^−^, K8/18^+^), and mature luminal (L2: CD49f^−^ EPCAM^+^, ER^+^, K8/18^+^) cells^8,10,51^. Significantly, the authors detected replicating KIT^+^ cells in all three main populations (Basal, L1, and L2), suggesting that each cluster may be maintained by its own KIT^+^ progenitor cell population, and proposed a continuous lineage hierarchy connecting the basal lineage to the two luminal branches via a bipotent MaSC. Furthermore, the authors highlight adult luminal cells that co-express both luminal (KRT8/18) and basal (KRT14) markers in situ.

The receptor tyrosine kinase KIT (c-Kit) has previously been identified as a defining marker of mammary epithelial progenitor cells (summarized in Table 1) and of the cells of origin of BRCA1-mutation breast cancer, luminal ER^−^ cells^17,28,34,40,50,55^. Similar to Nguyen et al.^51^, in Regan et al.^28^, we identified in the mouse, and also functionally tested via in vitro colony-forming assays and cleared mammary fat pad transplantation, c-Kit^−^ and c-Kit^+^ cell states within each of the mammary epithelial basal (CD24^+/Low^ Sca-1^−^ CD49f^+/High^ c-Kit^−^ and c-Kit^+^), myoepithelial (CD24^+/Low^ Sca-1^−^ CD49f^+/Low^ c-Kit^−^ and c-kit^+^), luminal ER^−^ (CD24^+/High^ Sca-1^−^ c-Kit^+/Low^ and c-Kit^+/High^), and luminal ER^+^ (CD24^+/Low^ Sca-1^−^ c-Kit^−^, CD24^+/Low^ Sca-1^+^ c-kit^−^ and c-kit^+^) cellular compartments^28^. The expression of *KIT*, as well as the luminal markers *KRT8/18* and *ESR1* and basal marker *KRT14*, in each of Nguyen et al.’s human breast populations of B, Myo, L1.1, L1.2, and L2, are consistent with the expression levels reported in Regan et al.^28^ in the corresponding murine basal, myoepithelial, luminal ER^–^ c-Kit^+/High^, luminal ER^−^ c-Kit^+/Low^, and luminal ER^+^ cells, respectively (Fig. 1). The KIT^+^ cells identified by Nguyen et al.^51^ are therefore likely equivalent to the c-Kit^+^ progenitor cells previously reported in Regan et al.^28^, which was the first study to functionally characterize c-Kit as a progenitor marker in the mammary gland (Table 1). When discussing KIT as a progenitor cell marker, Nguyen et al. incorrectly cite Stingl et al.^56^ and Shehata et al.^10^. These papers, respectively, did not investigate or functionally test c-Kit as a progenitor marker in the mammary gland.

**Table 1:** Studies demonstrating that luminal ER^−^ cells are enriched for c-Kit and that c-Kit identifies progenitor cells in the mammary epithelium^2,5,6,9,10,17,28,29,34,40,51–55,73,88–94^.

Study (year)	Method(s)	Cells/Progenitor cell marker(s)	Results
Natali et al. (1992)^88^ Matsuda et al. (1993)^89^ Hines et al. (1995)^90^ Ulivi et al. (2004)^91^ Tsuda et al. (2005)^92^ Westbury et al. (2009)^94^	Immunohistochemistry	Normal human breast tissue	High levels of c-Kit protein detected in the luminal alveolar/ductal epithelium but not in the basal/myoepithelial layer.
Shackleton et al. (2006)^2^ Stingl et al. (2006)^9^ Sleeman et al. (2006)^5^ Sleeman et al. (2007)^6^ Asselin-Labat et al. (2007)^29^	FACS Colony-forming assays Gland reconstitution Immunostaining Gene expression analysis	Mouse mammary cell populations Basal CD24^+/Low^ Sca-1^−^ CD49f/CD29^+/High^ Luminal ER^−^ CD24^+/High^ Sca-1^−^/CD61^+^	Luminal ER^−^ cells are in vitro progenitors and possess limited mammary gland repopulation potential. Basal cells contain facultative MaSCs.
Kendrick et al. (2008)^34^	Transcriptome analysis	Mouse mammary cell populations Basal CD24^+/Low^ Sca-1^−^ Luminal ER^−^ CD24^+/High^ Sca-1^−^	Luminal ER^−^ CD24^+/High^ Sca-1^−^ progenitor cells are enriched for c-Kit expression.
Lim et al. (2009)^40^ Lim et al. (2010)^17^	FACS Colony-forming assays Gland reconstitution Immunostaining Transcriptome analysis	Mouse mammary cell populations Basal CD29^hi^ CD24^lo^ CD61^+^ Luminal ER^−^ CD29^lo^ CD24^+^ CD61^+^ Human mammary cell populations Basal CD49f^+/hi^ EpCAM^+/lo^ Luminal ER^−^ CD49f^+^ EpCAM^+/hi^	c-Kit is highly expressed in mouse and human luminal progenitor cells. Functional testing of isolated c-Kit^+^ cells was not carried out in these studies.
Regan et al. (2012)^28^ [Epub 18 July 2011]	FACS Colony-forming assays Gland reconstitution Immunostaining Gene expression analysis	Mouse mammary cell subpopulations Basal CD24^+/Low^ Sca-1^−^ CD49f^+/High^ c-Kit^−^ Basal CD24^+/Low^ Sca-1^−^ CD49f^+/High^ c-Kit^+^ Luminal ER^−^ CD24^+/High^ Sca-1^−^ c-Kit^+/Low^ Luminal ER^−^ CD24^+/High^ Sca-1^−^ c-Kit^+/High^ Luminal ER^+^ CD24^+/High^ Sca-1^+^ c-kit^+^	c-Kit is an in vitro and in vivo functional marker of mammary progenitors and lineage-primed cell states in basal, luminal, ER^−^, and luminal ER^+^ cell populations. Facultative MaSCs are CD24^+/Low^ Sca-1^−^ CD49f^+/High^ c-Kit^−^.
Asselin-Labat et al. (2011)^55^ [Epub 19 Sept. 2011]	FACS Colony-forming assays Gland reconstitution Immunostaining Gene expression analysis	Mouse mammary cell subpopulations Luminal ER^−^ CD29^lo^ CD24^+^ CD14^+^ c-kit^−/lo^ Luminal ER^−^ CD29^lo^ CD24^+^ CD14^+^ c-kit^+^	c-Kit^+^ luminal cells expand during early pregnancy and are in vitro colony-forming progenitors. In vivo functional testing of isolated c-Kit^+^ cells was not carried out.
Shehata et al. (2012)^10^	FACS Colony-forming assays Gland reconstitution Immunostaining Gene expression analysis	Mouse mammary cell subpopulations Luminal ER^−^ EpCAM^+^ Sca-1^−^ CD49b^+^ CD14^+^ Luminal ER^+^ EpCAM^+^ Sca-1^+^ CD49b^+^ CD14^+^ Human mammary cell subpopulations Luminal CD49f^+^ EpCAM^+/hi^ ALDH^+^ ERBB3^+^ Luminal CD49f^+^ EpCAM^+/hi^ ALDH^−^ ERBB3^+^ Luminal CD49f^+^ EpCAM^+/hi^ ALDH^−^ ERBB3^−^	Identified luminal ER^−^ and luminal ER^+^ progenitor cells in mouse and human. Detected c-Kit^+^ cells in the luminal populations of FVB/N mice but not in C57Bl6/J mice. Functional testing of isolated c-Kit^+^ cells was not carried out in this study.
Pal et al. (2017)^52^	scRNA-Seq	Mouse mammary cell populations Basal CD29^hi^ CD24^+^ Luminal CD29^lo^ CD24^+^	Hierarchical clustering revealed that luminal progenitors are enriched for c-Kit. Transcriptome mapping identified rare c-Kit^+^ lineage-primed basal cells.
Bach et al. (2017)^53^	scRNA-seq	Nulliparous, embryonic, lactating, and post-involution mouse mammary cells EpCAM^+^	Identified c-Kit^+^ luminal progenitor cells that give rise to intermediate, alveolar, and hormone-sensitive progenitors.
Kim and Villadsen (2018)^93^	Immunohistochemistry	Normal human breast tissue EpCAM^+^ Ki-67^+^ KIT^+^	KIT^+^ cells constitute a proliferating (Ki-67^+^) luminal progenitor compartment during homeostasis of the resting gland.
Nguyen et al. (2018)^51^	scRNA-seq	Human mammary cell populations Basal (B) CD49f^High^ EPCAM^+^ Luminal (L1) ER^−^ CD49f^+^ EPCAM^+^ Luminal (L2) ER^+^ CD49f^−^ EPCAM^+^	Identified KIT^+^ progenitor cells in each mammary population, including L1.1 luminal (ER^−^ KIT^+/High^) and L1.2 luminal (ER^−^ KIT^+/Low^) progenitors.
Giraddi et al. (2018)^54^ Chung et al. (2019)^73^	scRNA-seq snATAC-seq	Embryonic and postnatal mouse mammary cells EpCAM^+^	c-Kit is most highly expressed and chromatin accessible in luminal progenitor cells.

**Fig. 1:**
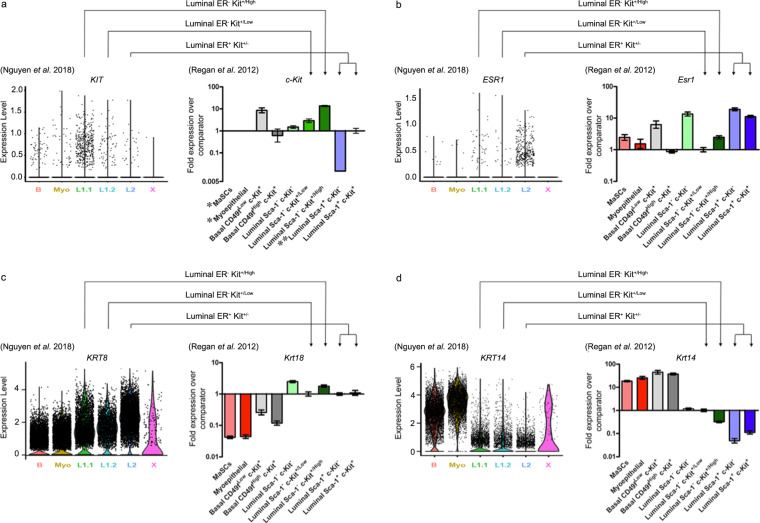
Comparison of gene expression in cell populations identified by Nguyen et al.^51^ and Regan et al.^28^. Nguyen et al.^51^ violin plots showing the expression pattern of progenitor marker *KIT* (**a** LHS), luminal genes *ESR1* and *KRT8* (**b**, **c** LHS), and basal gene *KRT14* (**d** LHS) grouped by final cluster determination in human mammary epithelium. B = basal (containing facultative MaSCs), Myo = myoepithelial. Regan et al.^28^ gene expression in the different cellular subpopulations as determined by qPCR for progenitor gene *c-Kit* (**a** RHS) relative to comparator luminal Sca-1^+^ c-Kit^+^ cells, luminal genes *Esr1* and K*rt18* (**b**, **c** RHS), and basal gene *Krt14* (**d** RHS) relative to comparator luminal Sca-1^−^ c-Kit^+/Low^ cells, in murine mammary epithelium. Data are presented as fold expression levels ±95% confidence intervals (*n* = three independently harvested isolates of each cell population). *Gene expression was undetectable in these populations in all three independent isolates. **Gene expression was only detected (at very low levels) in two of three isolates of the luminal Sca-1^+^ c-Kit^−^ population. Therefore, no error bars are shown for this sample. Images used with permission under a CC-BY 4.0 license from Nguyen et al.^51^ and Regan et al.^28^.

Nguyen et al.^51^ observed fractions of cells that co-express both luminal K8 and basal K14 markers, and report that such K8^+^ K14^+^ cells had previously been observed in mouse fMASCs by Spike et al.^37^ (such fetal cells were also previously described by Sun et al.^57^), but not in adult human tissue in homeostasis. However, while the canonical view among mouse mammary developmental biologists is that the K5/14 pair is a basal marker and the K8/18 pair is a luminal marker^58–60^, breast pathologists have known for many years that keratins 5 and 14 (and indeed another “basal” keratin, 17) are in fact expressed in basal cells of human breast ducts and in the luminal cells of the terminal ductal lobuloalveolar units (TDLUs)^58,61–64^. Indeed, K5/K18 and K14/K18 double-positive cells are not uncommon in human TDLUs^61^. More recently, Boecker et al.^65^ identified K5^+^ K18/19^−^ and K5^+^ K18/K19^+^ populations in the luminal layer of ductal and TDLU breast tissue in situ^65^, while in human breast epithelial populations isolated by flow cytometry, the progenitor populations (Lin^−^ CD49f^+^ EpCAM^hi^) include cells double-positive for K5/6 and K14 — and notably are also c-KIT^+^^40^. To add to the complexity of these marker patterns, K19 has been described both as a marker of progenitors^66–68^ and highly expressed in differentiated luminal ER^+^ cells^6,69^.

Boecker et al.^65^ termed the populations they identified as progenitors and intermediary cells, respectively, but it is difficult to definitively assign such functions purely on the basis of marker expression, or indeed ex vivo assays. Of course, human breast tissue cannot be lineage-traced through transgene activation as one can in the mouse, but use of cytochrome C oxidase (CCO) mutations in the mitochondrial genome has proven feasible as an approach. Cereser et al.^70^ report the presence of CCO-deficient clonal expansions in both ducts and TDLUs of normal breast^70^. Notably, the expansions were limited to the luminal layers, and they found no evidence of luminal CCO-deficient clones contributing to the basal layer. Therefore, if the K5/K14/c-KIT^+^ luminal cells of the human breast are indeed progenitors, they are lineage-restricted.

Keratin expression patterns in the mouse mammary epithelium are somewhat easier to define, but also not as straightforward as often suggested. Unlike in the human, when analyzed in situ, K14 and K8/18 in the mouse appear to be restricted to the basal and luminal cell layers, respectively. Indeed, we have rarely (if ever) observed a luminal cell in the normal resting adult mammary gland we could confidently say is K14 positive, or a basal cell that is K8/18 positive, by immunofluorescence in situ, and this is in agreement with most studies. However, immunohistochemical analysis of the mouse mammary gland by Mikaelian et al.^59^ has detected rare weak K14 staining of luminal cells from birth to puberty and weak K8/18 labeling of basal cells during mammary morphogenesis, which were most easily visualized during lactation^59^. As an added complication, it should be noted that in the mammary alveoli, the basal/myoepithelial cells form a classic “basket-like network” around the secretory cells, and in that location, the “luminal” cells are in fact touching the basement membrane through the gaps between the myoepithelial cells. Interestingly, therefore, in agreement with Mikaelian et al.^59^, when basal and luminal subpopulations were isolated by flow cytometry and stained by immunofluorescence, we found that c-Kit^+^ luminal cells (which were approx. 50% of the total mammary epithelium) were all strongly K18^+^ but also weakly K14^+^, and that c-Kit^+^ basal cells were strongly K14^+^ and weakly K18^+^ (Fig. 2b)^28^. c-Kit-negative single luminal and basal cells prepared and stained at the same time were respectively K18^+^ K14^−^ and K14^+^ K18^−^, suggesting that we were not seeing background staining in the c-Kit-positive cells. This discrepancy is likely due to signal/noise ratio when using in situ immunofluorescence approaches — enhancing the K14 staining to a level where it can be detected in luminal cells would result in a huge excess of staining from the basal cells as well as background signal from other cell types in the mammary gland (and likewise for K18 detection in basal cells), which is notorious for background fluorescence coming from adipocytes. Thus, only approaches based on single-cell separation will accurately detect mouse cells expressing the “luminal” keratin 18 and the “basal” keratin 14, and as we report using such approaches, such cells express the c-Kit marker^28^. Note that the scRNA-seq analysis of mouse mammary epithelium by Bach et al.^53^ shows that a subset of luminal cells have *Krt14* expression levels equivalent to the mean expression level of *Krt14* in basal cells. Their differentiation trajectory maps show that the Krt14-expressing luminal cells are enriched in a progenitor population that is also c-Kit-positive^53^.

**Fig. 2:**
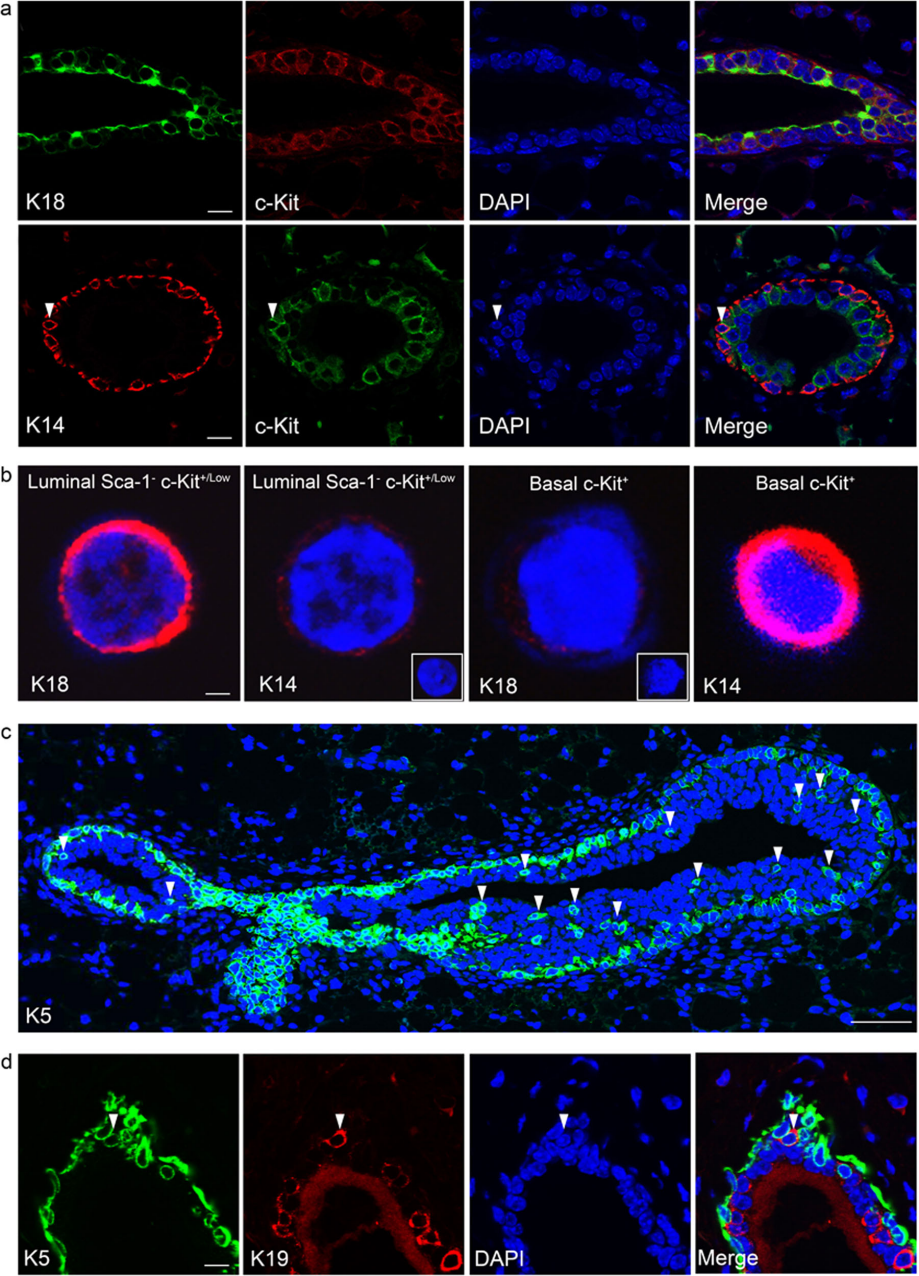
Basal and luminal marker expression suggests potential for differentiative plasticity in the mouse mammary gland in situ. **a** Immunofluorescence of sections though the mammary fat pads of adult virgin female FVB mice stained with antibodies against the luminal markers K18 and c-Kit and the basal marker K14. c-Kit staining is located predominantly in the K18^+^ K14^−^ luminal layer, although occasional K14^+^ c-Kit^+^ basal cells are detected (arrowhead). Bar = 40 µm. **b** K18 and K14 staining of freshly isolated single c-Kit^+^ luminal and c-Kit^+^ basal cells from adult virgin mice sorted directly onto slides. Insets show c-Kit^−^ luminal and basal cells negative for K14 (LHS) and K18 (RHS), respectively (bar = 3 µm). The numbers of cells examined and overall staining patterns are given in Table 1 of Regan et al.^28^. **c** Basal K5 staining in the terminal end buds (TEBs) and subtending duct of 4-week-old pubertal mouse mammary epithelium. K5 staining is located predominantly in the basal layer. Occasional K5^+^ cells are detected in the luminal layer (arrowheads). Bar = 40 μm. **d** Section through a cleared fat pad outgrowth double-stained for basal K5 and luminal K19. A K5^+^ K19^+^ double-positive cell is observed in the basal layer (arrowhead). Bar = 40 µm. All cells were counterstained with DAPI (blue).

In contrast, we find that cells double-positive for “basal” keratin 5 and “luminal” keratin 19 are readily detectable in the mouse luminal epithelium in situ (Fig. 2c, d). Interestingly, K19 has been proposed to be a neutral switch keratin that permits the changeover of one type of cytoskeleton to the other^68,71^. We have particularly noted K5-positive cells in the body cell region of terminal end buds in situ (Fig. 2c). The origin of these cells is unclear. Rios et al.^16^ reported that using a *Krt5*-promoter-driven cell-labeling approach, labeled cells were only observed in the basal compartment, but generated both luminal and basal daughter clones, and hence proposed the existence of bipotent basal stem cells arising from the basal layer of the TEBs^16^. However, the work of Scheele et al.^23^ and others^18–23,46,47^ suggests that cap cells (the basal cell layer of the TEBs) do not contribute to the luminal layer of the subtending duct; therefore K5-positive body cells, if they are cap cell-derived, are unlikely to contribute to outgrowth of the ducts. In contrast, if these cells are derived from the body cells, they are switching on high levels of K5 expression, but whether this is only transient — perhaps a temporary failure of lineage specification in a newly established daughter cell that is later corrected — is unclear.

Therefore, while use of keratins as basal/luminal lineage markers is more robust in the mouse mammary epithelium than in the human, single-cell analysis approaches have demonstrated that even the mouse has a more promiscuous pattern of keratin expression than previously suspected, and that this promiscuous expression of keratins is seen in c-KIT^+^ stem/progenitor cells. Plasticity in the expression of keratins and other genes within c-Kit^+^ luminal progenitors may relate to their potential to contribute to multiple cell lineages during epithelial remodeling, e.g., at involution of the mammary gland after weaning^72^. In addition, the phenotypic plasticity and multilineage differentiation potential of these luminal progenitors is consistent with their ability to give rise to tumors with basal features^40,50^, as well as lineage switching in response to injury and oncogene activation^20,24,49^. It is clear, therefore, that a great deal of caution must be used when keratin promoters are being used for lineage-tracing studies in the mouse or for assigning luminal/basal identity in human cells. Indeed, in a dissociated human breast epithelial cell population, keratin expression levels alone cannot be used to assign basal/luminal identity to a cell with any confidence.

To address the debate as to whether homeostasis and development in the postnatal mammary gland are maintained by bipotent MaSCs^15,16,43^ or lineage-restricted basal and luminal cells^54,19–22^, Nguyen et al.^51^ performed pseudotemporal reconstruction-based lineage hierarchy analysis. This analysis identified a continuous lineage connecting the basal lineage, via a bipotent MaSC, to the two luminal branches. These results agree with previous models of mammary differentiation wherein a bipotent basal MaSC generates daughter cells that differentiate into myoepithelial and luminal cell lineages^15,16,43^. However, Nguyen et al. propose that their results differ from previous studies in that L1.2 cells (luminal ER^−^ c-kit^+/Low^ cells) are progenitors to L1.1 cells (luminal ER^−^ c-Kit^+/High^ cells), and that c-Kit^+/High^ L1.1 cells are another type of mature differentiated luminal cell rather than a luminal progenitor upstream of luminal ER^+^ L2 cells. Based on this pseudotemporal analysis, the authors suggest that KIT is not a marker of luminal progenitor cells. This is a surprising conclusion considering that L1.2 progenitor cells do express *KIT* (Fig. 1), which as well as being a defining marker of mouse and human progenitor cell gene expression signatures^17,34,40,52–54,73^, has been functionally demonstrated as a progenitor cell marker^28^ (Table 1).

Similar to Nguyen et al.^51^, Pal et al.^52^ used scRNA-seq to identify lineage relationships in the mouse mammary gland, and also suggested that bipotent basal MaSCs give rise to basal and luminal lineages^52^. Supporting our previous assessment of intermediate cells in the luminal lineage^28^, the authors also described the identification of intermediate luminal cells. Significantly, Pal et al. report the identification of rare mixed-lineage or “lineage-primed” c-Kit-expressing basal cells in the adult mammary gland and state, “It is presumed that these cells represent a transient population that is poised for commitment to the luminal lineage, reminiscent of “lineage-primed” stem and progenitor cells initially reported in the hematopoietic system.” These lineage-primed c-Kit^+^ basal cells comprised ~5% of the basal compartment and expressed luminal genes such as *Esr1, Prlr, Csn2*, and *Areg* in addition to basal genes. Pal et al. state, “these data suggest that the basal state may precede commitment to a luminal cell fate in the post-natal mammary gland.”

In Regan et al.^28^, we also identified cells that we described as lineage-primed basal cells (CD24^+/Low^ Sca-1^−^ CD49f^+/High^ c-Kit^+^) in the adult mammary gland that expressed luminal genes, including those described by Pal et al. (*Esr1, Prlr, Csn2*, and *Areg*), but that clustered with the basal facultative MaSCs^28^. Significantly, we functionally tested these cells by single-cell cleared mammary fat pad transplantation and demonstrated that they can reconstitute an entire ductal tree, although at a lower frequency (1 in 8 ± 95% CI 1 in 3–1 in 21.3) than facultative c-Kit^−^ MaSCs (1 in 3 ± 95% CI 1 in 1.69–1 in 6.27), the highest enrichment of facultative MaSCs reported to date and potentially a pure facultative MaSC population. Based on these data, we came to the same conclusion as Pal et al.^52^ and described these c-Kit^+^ basal cells as intermediate MaSCs that were undergoing “lineage priming,” in which stem cells express genes associated with their differentiated daughter populations^74,75^. This was the first time that lineage-primed basal cells in the adult mammary gland had been reported and functionally tested.

In contrast to Nguyen et al.^51^ and Pal et al.^52^, scRNA-Seq by Bach et al.^53^ on mouse mammary epithelial cells at nulliparous, mid gestation, lactation, and post involution concluded that, rather than clearly defined clusters maintained by their own stem/progenitor population, a continuous spectrum of differentiation exists. In this model, a common luminal progenitor cell, which notably expressed c-Kit at high levels, gives rise to intermediate, restricted alveolar, and hormone-sensitive progenitors.

More recently, Giraddi et al.^54^ used scRNA-seq and transposase-accessible chromatin sequencing (ATAC-seq), which examines global chromatin accessibility^76^ of embryonic, postnatal, and adult mouse mammary epithelia, to elucidate the lineage hierarchies and biological programs that generate mature cell types from their embryonic precursors^54^. This work was more consistent with the conclusions of Bach et al.^53^ than Nguyen et al.^51^ and Pal et al.^52^, as well as the lineage-tracing studies showing that while embryonic mammary cells are bipotent, in the adult gland, basal and luminal cell lineages are derived from and maintained by separate lineage-committed progenitor populations^18–24,42,46–48^.

Similar to Pal et al.^52^, Giraddi et al.^54^ also identified rare c-Kit^+^ basal cells, although they did not occur at a frequency greater than the expected doublet frequency (∼1%) of the 10X Genomics Chromium System sequencing platform^54^, a frequency similar to the c-Kit^+^ basal cells that Pal et al.^52^ also detected using the 10X platform. In contrast, the lineage-primed c-Kit^+^ basal cells that we identified in our 2012 study were visually confirmed to be single cells prior to performing the single-cell transplants, in which they displayed a transplantation-frequency intermediary to facultative c-Kit^−^ MaSCs and c-Kit^+^ luminal progenitor cells. In addition, immunofluorescence staining of single c-Kit^+^ basal cells demonstrated that they expressed both K14 and K18 (Fig. 2b)^28^.

Transcriptional profiling by Giraddi et al.^54^ did not detect any distinct adult basal stem cell subpopulation. However, ATAC-seq revealed that adult basal cells display an embryonic MaSC-type chromatin accessibility at luminal gene loci, which the authors speculate allows for lineage plasticity^54,73,77^. Such plasticity may account for acquisition of multilineage potential upon perturbation of a homeostatic niche environment, such as during cell isolation and ex vivo culture, transplantation assays, wounding, and cancer^49,54,77–80^. The performance of a particular cell type during functional assays may therefore be a product of both their transcriptional heterogeneity and the context in which they are challenged^49^. Similar functional stem cell capacities have also been described in embryonic tissue, intestine, bone marrow, skin, and lung^81–83^. These observations challenge the concept of fixed-cell identities in complex tissues, and suggest a more fluid concept of cell state (for a more detailed discussion of this concept see Wahl and Spike^49^). With this in mind, a potential mammary epithelial cell hierarchy based on lineage tracing, functional analyse, and recent scRNA-seq and snATAC-seq studies is shown in Fig. 3.

**Fig. 3:**
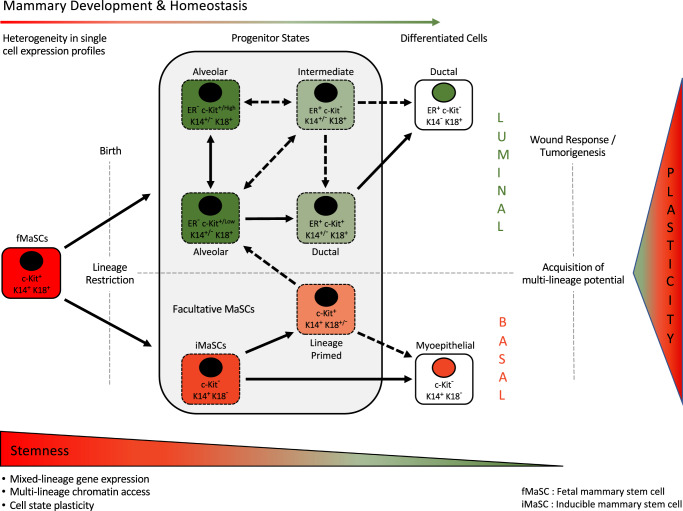
Proposed model (adapted with permission from Giraddi et al.^54^) of the mammary epithelial cell-state lineage hierarchy in the postnatal gland based on lineage tracing, functional assays, scRNA-seq, and snATAC-seq. Bipotent fetal mammary stem cells (fMaSCs) are present in the embryo and become lineage-restricted after birth. In the adult gland, each lineage is maintained by its own c-Kit^+^ progenitor. Loss of homeostasis (e.g., injury, cell isolation, ex vivo culture, and transplantation) or tumorigenesis may trigger a wound response that leads to acquisition of multilineage potential by facultative inducible MaSCs (iMaSCs), c-Kit^+^ lineage-primed, and progenitor cell states. Lineage-primed c-Kit^+^ basal cells that express intermediate levels of luminal genes may represent a transient or intermediate population that precedes commitment to the luminal lineage^28,52^. Gene expression analysis suggests that an alternative route for generating ER^+^ cells from intermediate luminal cell states may also exist.

Future studies that aim to map fluid cell-state dynamics and their regulatory mechanisms will require the use of single-cell and single-molecule epigenomic technologies that reveal a cell’s regulatory potential, rather than its current state, as indicated by its transcriptome^84,85^. Indeed, Chung et al.^73^ recently demonstrated that single-cell chromatin accessibility mapping of mammary gland development using single-nucleus ATAC-seq (snATAC-seq) enables greater resolution of cell-state heterogeneity, and to be a better indicator of cell state during development than scRNA-seq^73^. The lineage relationships delineated in this study were consistent with those of Bach et al.^53^ and Giraddi et al.^54^, and also found *c-Kit* to be most highly expressed and chromatin accessible in luminal progenitor cells.

## Concluding remarks

Taken together, the weight of evidence supports c-Kit as a progenitor marker in the mammary epithelium and, more importantly, one that is functionally characterized and can be used to enrich stem/progenitor cells. Indeed, we have already begun to understand the signaling pathways downstream of c-Kit in mammary progenitor cells^86^. scRNA-seq studies, which allow for comprehensive and unbiased analysis of the different cell types that constitute a heterogeneous tissue^87^, have been extremely valuable in contributing to our understanding of lineage relationships and cell-state heterogeneity in the mammary gland. However, in order to fully understand the significance of these studies, it is essential to link them to functional data, in particular where such data already exist, and future studies should aim to do so. The evidence from lineage tracing, scRNA-seq, and snATAC-seq studies currently supports a model in which fMaSCs in the embryo are bipotent, whereas in the adult gland, stem/progenitor cells are lineage-restricted, and facultative MaSCs (defined by functional studies) are induced to acquire multilineage potential upon loss of homeostasis/injury. Bipotent fetal MaSCs are described as fMaSCs to differentiate them from adult facultative MaSCs. However, the scientific literature up to now continues to refer to adult cells with facultative stem cell potential simply as MaSCs or, in a handful of publications, adult MaSCs (aMaSCs)^37,49^, which is no longer an accurate or apt description. We therefore propose the renaming of MaSCs in the postnatal gland as “inducible mammary stem cells” (iMaSCs). This new definition will help to more clearly define the status and stem cell potential of functionally defined iMaSCs in the era of large-scale single-cell molecular profiling.

## Data Availability

Source data for all figures and tables are provided in the paper. No new datasets have been generated or analyzed for this article.
